# Anti-Ma-associated paraneoplastic cerebellar degeneration in a patient with nodular lymphocyte-predominant Hodgkin lymphoma: a case report

**DOI:** 10.1186/s12883-020-01929-4

**Published:** 2020-09-23

**Authors:** Ryoma Inui, Kenki Saito, Yoshimitsu Shimomura, Daisuke Yamashita, Michi Kawamoto, Takayuki Ishikawa

**Affiliations:** 1 Department of Neurology, 2-1-1 Minatojima-Minamimachi, Chuo-ku, Kobe, 650-0047 Japan; 2 Department of Hematology, 2-1-1 Minatojima-Minamimachi, Chuo-ku, Kobe, 650-0047 Japan; 3 Department of Pathology, Kobe City Hospital Organization Kobe City Medical Center General Hospital, 2-1-1 Minatojima-Minamimachi, Chuo-ku, Kobe, 650-0047 Japan

**Keywords:** Anti-Ma2, Nodular lymphocyte-predominant Hodgkin lymphoma, Paraneoplastic cerebellar degeneration

## Abstract

**Background:**

Paraneoplastic cerebellar degeneration (PCD) is a devastating paraneoplastic syndrome that occasionally occurs in patients with Hodgkin lymphoma (HL). Anti-Ma2 is a well-characterized onconeuronal antibody and one of the causes of PCD. There has been only one previous report of anti-Ma2-associated paraneoplastic syndrome as a complication of HL. Here we present a rare case of anti-Ma2-associated PCD in a patient with nodular lymphocyte-predominant HL (NLPHL).

**Case presentation:**

A 77-year-old man with a 3-month history of gait instability and a 2-month history of oscillopsia was referred to our hospital for further investigation. On examination, his cognition was normal. He had nystagmus in all directions of gaze; specifically, he had horizontal and rotatory nystagmus in the primary position, downbeat nystagmus after right, left, and up gaze, and upbeat nystagmus after down gaze. Although his limb ataxia was mild, his trunk ataxia was so pronounced that he was unable to walk without support. We strongly suspected paraneoplastic syndrome and tested for neuronal autoantibodies. The anti-Ma2 antibody was strongly positive in the blood and cerebrospinal fluid but other antineuronal autoantibodies were negative. Computed tomography showed an enlarged lymph node in the right axilla but no masses. Biopsy confirmed a diagnosis of NLPHL. The NLPHL cells stained with anti-Ma-2 antibody in the cytoplasm, suggesting these abnormal cells contained protein that was cross-reactive with Ma-2.

**Conclusions:**

To the best of our knowledge, this is the first case of anti-Ma2-associated PCD in a patient with NLPHL that was confirmed using immunostaining of the lymph node tissue with anti-Ma2 antibody. Our case confirms an association between anti-Ma2-associated PCD and NLPHL.

## Background

Paraneoplastic cerebellar degeneration (PCD) is a devastating paraneoplastic syndrome (PNS) characterized by subacute cerebellar ataxia, dysarthria, and ocular dysmetria, which is preferentially associated with ovarian, breast, small cell lung cancer, and Hodgkin lymphoma (HL) [1, 2]. Most cases of PCD are considered to be immune-mediated by onconeural protein and its antibodies. Three antibodies, anti-Yo, anti-Tr, and anti-metabotropic glutamate receptor 1 (mGluR1), are predominantly associated with PCD, and several other antibodies, including anti-Hu, anti-Ma1, and anti-Ma2, have been reported in association with a variety of neurologic syndromes [2–4]. Anti-Tr and anti-mGluR1 have been reported in patients with HL [5–10].

The Ma2 antigen is selectively expressed in neurons. Anti-Ma2-associated PNS includes limbic, hypothalamic, brainstem encephalitis, and cerebellar degeneration [11]. According to the diagnostic criteria published by the European Federation of Neurological Societies/Peripheral Nerve Society, anti-Ma2 is a well-characterized onconeuronal antibody and a definitive diagnosis of PNS can be made in a patient with this antibody [3, 4]. Anti-Ma2-associated PNS occurs mainly in patients with underlying malignancy and has been associated with testicular cancer and lung cancer [11]. There has been only one previous report of anti-Ma2-associated PNS as a complication of HL [12].

Herein, we present a rare case of anti-Ma2-associated PCD in a patient with nodular lymphocyte-predominant HL (NLPHL) in whom the association between anti-Ma2 and NLPHL was confirmed by lymph node immunostaining using the anti-Ma2 antibody for the first time.

## Case presentation

A 77-year-old man with a 3-month history of gait instability and a 2-month history of oscillopsia was referred to our hospital for further investigation. On examination, his cognition was normal. He had nystagmus in all directions of gaze, specifically horizontal and rotatory nystagmus in the primary position, downbeat nystagmus after right, left, and up gaze, and upbeat nystagmus after down gaze. Although his limb ataxia was mild, his trunk ataxia was so severe that he was unable to walk without support. Manual muscle testing was 5/5 in the upper and lower extremities bilaterally. He had right axillary lymphadenopathy but no splenomegaly or hepatomegaly.

**Fig. 1:**
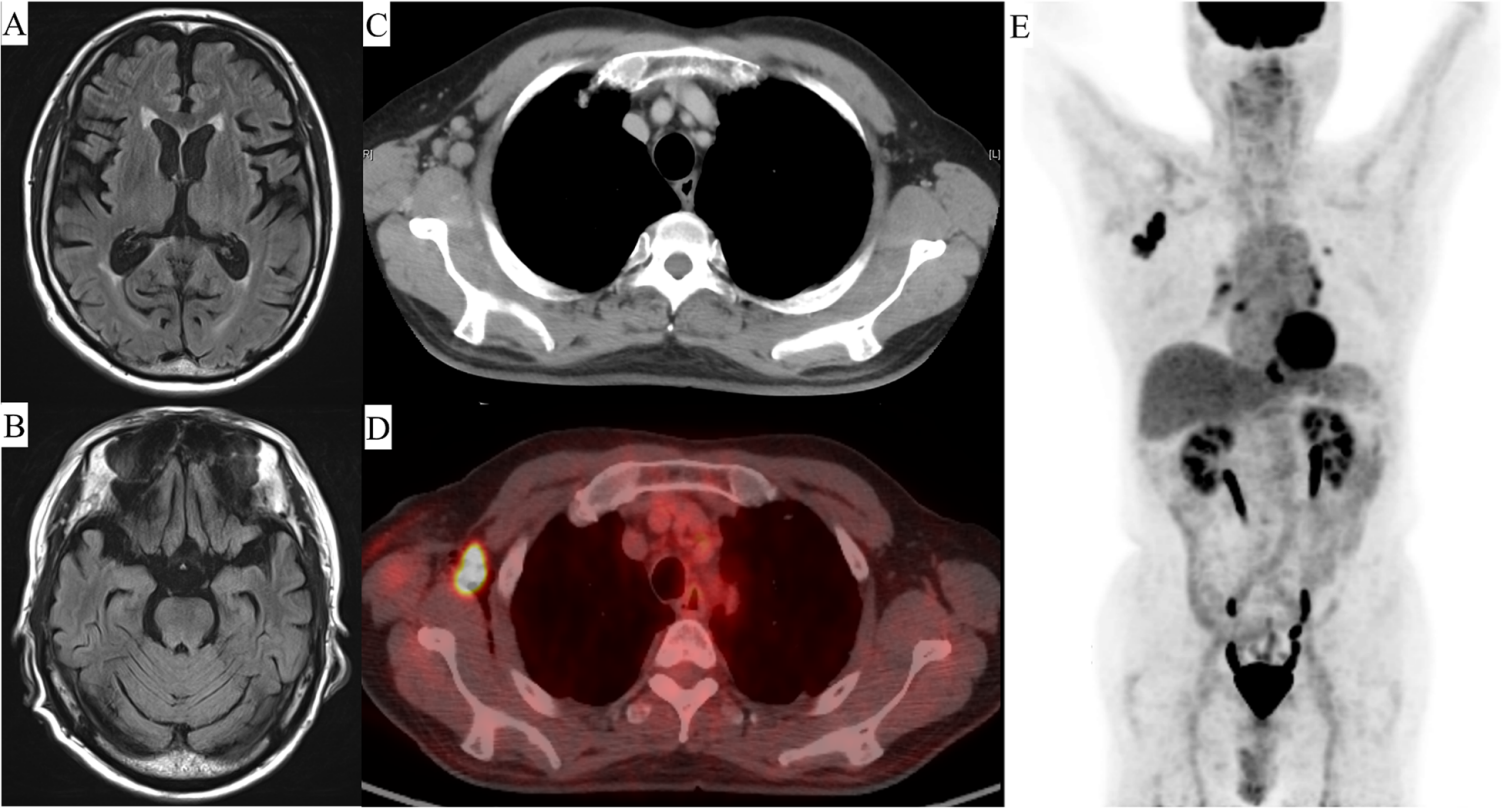
Image findings. **a** Magnetic resonance imaging on admission to hospital reveals no parenchymal signal change. **b** Computed tomography (CT) before treatment reveals an enlarged lymph node in the right axilla (1.9 × 2.2 mm). **c** ^18^F-fluorodexyglucose (FDG)-positron emission tomography (PET)/CT shows limited FDG uptake (maximum standardized uptake value, 29.13). **d** ^18^F-FDG-PET/CT shows no FDG uptake other than in the lymph node in the right axilla

Laboratory data on admission revealed the following: hemoglobin, 13.6 g/dL; platelets, 173 × 10^9^/L; white blood cell count, 5.1 × 10^9^/L; lactate dehydrogenase, 164 U/L; soluble interleukin 2 receptor, 365; erythrocyte sedimentation rate, 22; and antinuclear antibody, 1:320. Examination of the cerebrospinal fluid revealed no white blood cells, slight elevation of protein (57 mg/dL), presence of oligoclonal bands, and absence of myelin basic protein. A magnetic resonance scan of the brain did not show a signal change in the parenchyma (Fig. 1a, b).

We strongly suspected paraneoplastic syndrome and tested for neuronal autoantibodies. The anti-Ma2/Ta antibody was strongly positive in the blood (3+) and cerebrospinal fluid (2+) but other antineuronal autoantibodies (Hu, Ri, Yo, Tr, CV2/CRMP5, amphiphysin, recoverin, SOX1, titin, zic4, and GAD65) were negative.

Next, we performed computed tomography (CT) to identify the primary tumor, which showed an enlarged lymph node (1.9 × 2.2 mm) in the right axilla (Fig. 1c). ^18^F-fluorodeoxyglucose-positron emission tomography/CT showed limited fluorodeoxyglucose uptake (maximum standardized uptake, 29.13) in the same location (Fig. 1d, e) but no other mass.

**Fig. 2:**
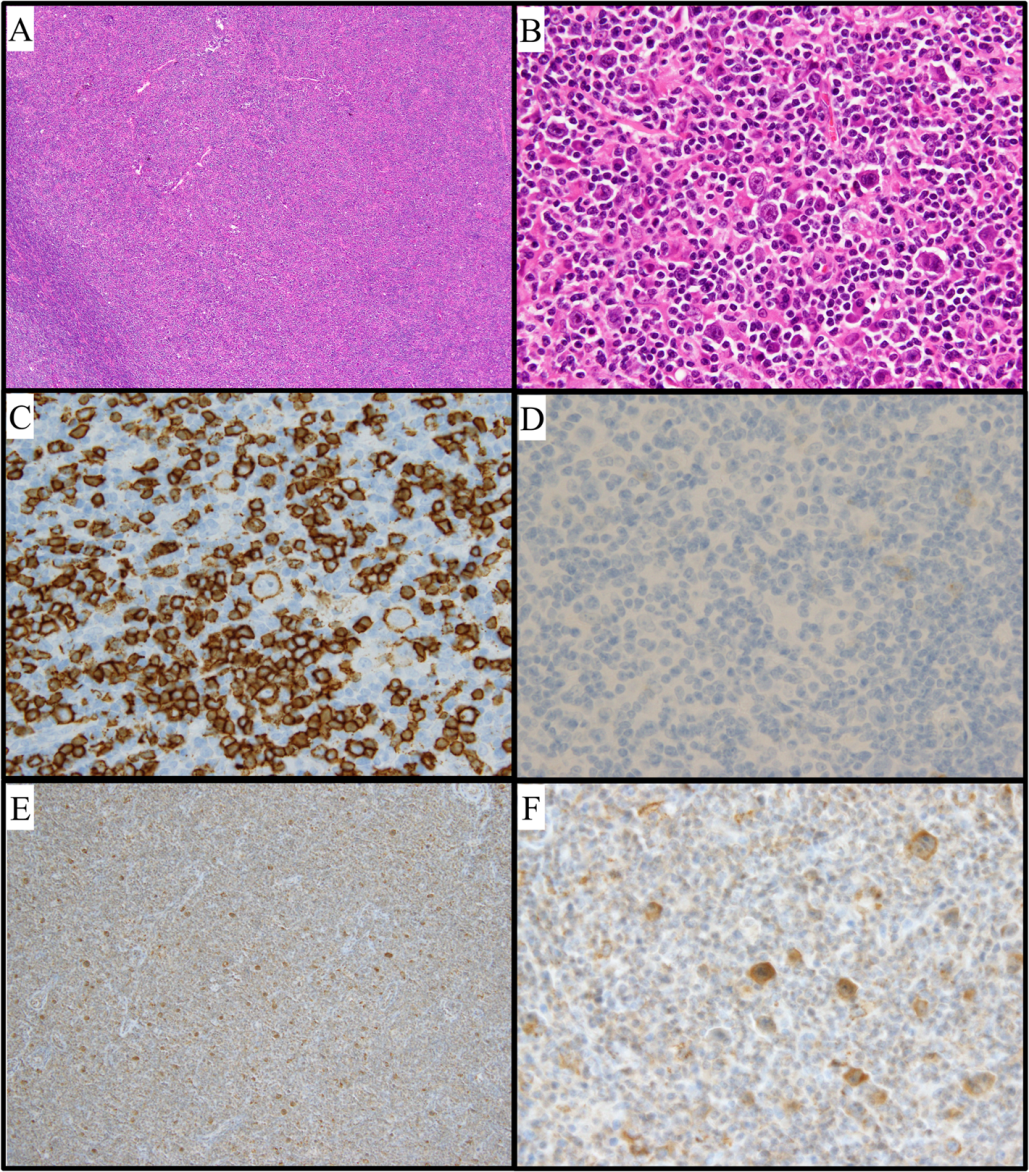
Histological findings for a biopsy specimen. **a** Hematoxylin-eosin (HE) staining, × 40 magnification. The lymph node structure almost completely effaced by a diffuse infiltrate. **b** HE staining, × 400 magnification. A scattered proliferation of large atypical cells found; nodular lymphocyte-predominant Hodgkin lymphoma diagnosed. **c** Immunostaining for cluster of differentiation (CD) 20, × 400 magnification. **d** Immunostaining for CD30, × 400 magnification. Tumor cells positive for CD20 and negative for CD30. **e** Immunostaining for anti-Ma2, × 40 magnification. Tumor cells stained for anti-Ma2. **f** Immunostaining for anti-Ma2, × 400 magnification. The cytoplasm of the tumor cells stained for anti-Ma2

Histopathology showed scattered proliferation of large atypical cells, which were positive for cluster of differentiation (CD)20 and BCL-6 and negative for CD10 and CD30 (Fig. 2a–d). There were small cells rosetting around the tumor cells, which were positive for CD3 and PD-1. The tumor cells and rosetting cells were surrounded by fibrotic tissue. These findings were consistent with NLPHL, pattern A, Ann Arbor stage I. Furthermore, the large atypical cells stained with anti-Ma-2 antibody (Abcam, Cambridge, UK) in the cytoplasm (Fig. 2e, f), suggesting the abnormal cells contained protein that was cross-reactive with Ma-2.

At the time of diagnosis of NLPHL and PCD, the patient had a SARA (Scale for the Assessment and Rating of Ataxia [13]) score of 16. We initiated R-CHOP (rituximab 375 mg/m^2^, cyclophosphamide 750 mg/m^2^, doxorubicin 50 mg/m^2^, vincristine 1.4 mg/m^2^, and prednisolone 60 mg/m^2^ for 5 days) and high-dose intravenous immunoglobulin (400 mg/kg for 5 days) in the expectation of an effect of rituximab and high-dose immunoglobulin on paraneoplastic syndrome [14, 15]. The patient’s neurologic symptoms gradually improved after initiation of R-CHOP. After the second cycle of R-CHOP, CT indicated a near complete response (Fig. 1d), and no oligoclonal bands were detected in the cerebrospinal fluid, which was also considered evidence of a therapeutic effect. Therefore, we added limited field radiation (39.6 Gy/22 fr) and weekly rituximab monotherapy (375 mg/m^2^, 6 cycles, total of 8). His symptoms improved to a maximum SARA score of 9.

## Discussion and conclusions

This report describes a patient with anti-Ma2-associated PCD as a complication of NLPHL. He was diagnosed to have PCD based on his symptoms and the presence of the anti-Ma2 antibody in the blood and cerebrospinal fluid. He was also diagnosed to have NLPHL on pathologic examination of a lymph node biopsy specimen. Histopathologic analysis also showed that the NLPHL cells stained for anti-Ma2 antibody in the cytoplasm. These findings confirmed the association between anti-Ma2-associated PCD and NLPHL.

Ma2 is selectively expressed intracellularly in neurons and tumors. The anti-Ma2 antibody categorized in PNS-related onconeuronal antibodies so-called well-characterized onconeuronal antibodies [3, 4]. Generally, a definitive diagnosis of PNS can be made in a patient with well-characterized onconeuronal antibodies even if no tumor is found [3]. However, the possibility of a synchronous primary cancer should be considered if the patient has a well-characterized onconeuronal antibody and a tumor for which there is no reported association with that antibody [3]. The patient may have another cancer that developed concurrently or after the diagnosis of PNS; therefore, additional testing is recommended. Our case was positive for anti-Ma2 antibodies in the blood and cerebrospinal fluid, but there had been only one previous report of anti-Ma2-associated paraneoplastic syndrome as a complication of HL. We could not detect any mass other than the lymph node in the right axilla, which was diagnosed as NLPHL by biopsy and found that the cytoplasm of NLPHL cells were stained by anti-Ma2 antibody, similar to some other cancers [11, 16]. These findings confirmed the association between NLPHL and anti-Ma2-associated PCD.

PCD occurs with HL in less than 1% of all HL cases [1, 2, 6, 7]. Anti-Tr antibodies are mainly associated with PCD as a complication of HL [7, 8]. A possible association between antibodies against mGLuR1 and HL has been reported, but anti-Ma2-associated PCD is rare in patients with HL [9, 10]. There has been one case report of anti-Ma2-associated PNS as a complication of HL [12]. The patient in that report presented with neuropsychiatric symptoms, cataplexy, hypoglycemic episodes, and a vertical supranuclear gaze palsy. His neurologic symptoms, except for the vertical supranuclear gaze palsy, improved after treatment of the HL, and all the symptoms improved after subsequent immunotherapy. The symptoms in our patient were different in that he had truncal ataxia and nystagmus in all directions of gaze and was diagnosed with PCD. According to a previous report on the clinical epidemiology and characteristics of anti-Ma2-associated PNS [11], patients with anti-Ma2-associated PNS as a complication of HL can develop a variety of symptoms. Therefore, we should screen for anti-Ma2 regardless of the symptomatology if PNS is suggested in a patient with HL.

The neurologic outcome of PCD is often poor, and most patients do not show improvement in the neurologic function [2]. Treatment of the underlying tumor is considered essential for neurologic stabilization, and immunotherapy, including plasma exchange, intravenous immunoglobulin, corticosteroids, and rituximab, is often attempted individually or in combination [2, 11, 15]. However, NLPHL has an indolent clinical course, and the 10-year overall survival rate is over 70% [17]. The standard treatment for NLPHL is radiation therapy for limited disease and immunochemotherapy, including R-CHOP, for advanced disease [18]. In our case, we added R-CHOP to radiation therapy as initial immunotherapy for PCD concurrently with treatment of the underlying disease because of the rapid progression of his neurologic symptoms. His neurologic findings showed slight improvement.

To the best of our knowledge, this is the first case of anti-Ma2-associated PCD in a patient with NLPHL, which was confirmed by lymph node immunostaining using anti-Ma2 antibody. Our case confirms the association between anti-Ma2-associated PCD and NLPHL. In addition, it suggests that PNS-related onconeuronal antibodies including anti-Ma2 should be screened regardless of a past history of HL if patients developed cerebellar degeneration as well as limbic condition, hypothalamitis or brainstem encephalitis.

## Data Availability

All data analyzed during this study are included in this manuscript.

## Abbreviations

CD: cluster of differentiation
CT: computed tomography
^18^F-FDG PET/CT: ^18^F-fluorodexyglucose-positron emission tomography/CT
HL: Hodgkin lymphoma
mGluR1: metabotropic glutamate receptor 1
NLPHL: nodular lymphocyte-predominant Hodgkin lymphoma
PCD: paraneoplastic cerebellar degeneration
PNS: paraneoplastic syndrome
SARA: Scale for the Assessment and Rating of Ataxia
